# Pathogenicity of Proteinase 3-Anti-Neutrophil Cytoplasmic Antibody in Granulomatosis With Polyangiitis: Implications as Biomarker and Future Therapies

**DOI:** 10.3389/fimmu.2021.571933

**Published:** 2021-02-18

**Authors:** Jérôme Granel, Brice Korkmaz, Dalila Nouar, Stefanie A. I. Weiss, Dieter E. Jenne, Roxane Lemoine, Cyrille Hoarau

**Affiliations:** ^1^ Université de Tours, Plateforme B Cell Ressources (BCR) EA4245, Tours, France; ^2^ Service d’Immunologie Clinique et d’Allergologie, Centre Hospitalier Régional Universitaire, Tours, France; ^3^ INSERM, Centre d’Etude des Pathologies Respiratoires (CEPR), UMR 1100, Tours, France; ^4^ Comprehensive Pneumology Center, Institute of Lung Biology and Disease, German Center for Lung Research (DZL) Munich and Max Planck Institute of Neurobiology, Planegg-Martinsried, Germany

**Keywords:** anti-neutrophil cytoplasmic antibodies, proteinase 3, granulomatosis with polyangiitis, pathogenicity, human neutrophils, biomarkers, new therapies

## Abstract

Granulomatosis with polyangiitis (GPA) is a rare but serious necrotizing auto-immune vasculitis. GPA is mostly associated with the presence of Anti-Neutrophil Cytoplasmic Antibody (ANCA) targeting proteinase 3 (PR3-ANCA), a serine protease contained in neutrophil granules but also exposed at the membrane. PR3-ANCAs have a proven fundamental role in GPA: they bind neutrophils allowing their auto-immune activation responsible for vasculitis lesions. PR3-ANCAs bind neutrophil surface on the one hand by their Fab binding PR3 and on the other by their Fc binding Fc gamma receptors. Despite current therapies, GPA is still a serious disease with an important mortality and a high risk of relapse. Furthermore, although PR3-ANCAs are a consistent biomarker for GPA diagnosis, relapse management currently based on their level is inconsistent. Indeed, PR3-ANCA level is not correlated with disease activity in 25% of patients suggesting that not all PR3-ANCAs are pathogenic. Therefore, the development of new biomarkers to evaluate disease activity and predict relapse and new therapies is necessary. Understanding factors influencing PR3-ANCA pathogenicity, *i.e.* their potential to induce auto-immune activation of neutrophils, offers interesting perspectives in order to improve GPA management. Most relevant factors influencing PR3-ANCA pathogenicity are involved in their interaction with neutrophils: level of PR3 autoantigen at neutrophil surface, epitope of PR3 recognized by PR3-ANCA, isotype and glycosylation of PR3-ANCA. We detailed in this review the advances in understanding these factors influencing PR3-ANCA pathogenicity in order to use them as biomarkers and develop new therapies in GPA as part of a personalized approach.

## Highlights

- Despite recent advances, GPA is still a serious disease with a high risk of relapse which is inconsistently predicted based on PR3-ANCA level alone.
- PR3-ANCAs have a pathogenic role in GPA: their binding with neutrophils by their Fab (on membrane-bound PR3) and Fc fragments (on Fc*γ*R) leads to auto-immune activation of neutrophils.
- Correlation between disease activity and circulating PR3-ANCA level is inconsistent suggesting that not all PR3-ANCAs are pathogenic.
- Different factors (paratope and glycosylation) influencing PR3-ANCA pathogenicity need to be taken into account to develop new biomarkers and therapies.

## Introduction

Granulomatosis with polyangiitis (GPA), formerly known as Wegener’s disease, is a form of necrotizing auto-immune vasculitis affecting predominantly small to medium vessels with histological inflammatory lesions and granulomas (1). GPA is relatively rare with an incidence rate of 10 to 20 new cases per million inhabitants per year and a prevalence between 120 and 140 cases per million inhabitants in Europe and the United States (2). This prevalence continues to increase (3). Its manifestations vary but mainly develop in the ear–nose–throat region (ENT), in the lungs and kidneys resulting in a necrotizing sinusitis, pulmonary capillaritis sometimes with alveolar hemorrhage and glomerulonephritis, all of which can be severe (2).

In vasculitis nomenclature, GPA is part of a group of anti-neutrophil cytoplasmic antibody (ANCA) associated vasculitis (AAV), along with eosinophilic granulomatosis with polyangiitis (EGPA) and microscopic polyangiitis (MPA) (1). AAVs are classified by the antigen recognized by ANCA: either proteinase 3 (PR3-ANCA) or myeloperoxidase (MPO-ANCA) (4). PR3 is a neutrophilic serine protease contained in neutrophil granules but also exposed at the membrane (5). The high-quality immunoassays are now used as the primary screening method for patients suspected of AAV, and immunofluorescence has been abandoned (4). Concerning GPA, PR3-ANCAs are found in about three quarters of patients and appear to be very specific (specificity > 90%) (6–8). In contrast, only 10% of GPA patients have MPO-ANCA, and less than 10% have no detectable ANCA (6). PR3-ANCA can be found in other conditions than AAV (9). Furthermore, PR3-ANCA can be found in healthy people (10, 11). But these natural antibodies to PR3 are only detected when samples are prepared (10). However, PR3-ANCAs have a direct pathogenic role in the disease. Indeed, PR3-ANCAs bind neutrophils allowing their auto-immune activation which is responsible for vasculitis lesions in GPA (12, 13).

According to recommendations from the European League Against Rheumatism (EULAR), the current initial management of patients with GPA involves the use of long-term immunosuppressive therapy, such as glucocorticoids, cyclophosphamide and, more recently, monoclonal antibodies as anti-CD20 (14). The total duration of these treatments after obtaining remission is at least two years (14). Despite treatment, GPA is a serious disease with an important mortality rate of 21.5% at five years when renal involvement is present (15), a significant morbidity related to the disease and its management (16) and a high risk of relapse of 30 to 50% within five years (17). Furthermore, the correlation between PR3-ANCA level, currently used for relapse management, and disease activity is inconsistent in the literature (18–23) except to predict relapse in patients with renal involvement (24, 25) or following treatment with rituximab (26, 27). Moreover, PR3-ANCA can persist in GPA patients during remission without predicting relapse (19, 22). Therefore, the development of new biomarkers to evaluate disease activity and predict relapse and new therapies is necessary.

PR3-ANCAs have a fundamental role in GPA by inducing auto-immune activation of neutrophils (12, 13). Therefore, understanding factors influencing PR3-ANCA pathogenicity, *i.e.* their potential to induce auto-immune activation of neutrophils, is necessary to develop new biomarkers to improve prediction of relapse and to develop new more specific therapies.

The aim of this review is to provide an overview of the advances in the understanding of the pathogenicity of PR3-ANCA in order to exploit them to develop new biomarkers and therapies in GPA. First, the importance of PR3-ANCA and neutrophils in the pathophysiology of the disease is discussed, then factors involved in the mechanism of auto-immune activation of neutrophils by PR3-ANCA are detailed and finally the understanding of these factors is examined to highlight avenues for the development of new biomarkers and therapies.

## Granulomatosis With Polyangiitis Pathophysiology: Arguments for a Pathogenic Role of Proteinase 3-Anti-neutrophil cytoplasmic Antibody

The role of PR3-ANCA in the pathophysiology of GPA has been studied widely since their discovery. In 1982, it was demonstrated that autoantibodies in sera of patients with segmental necrotizing glomerulonephritis stained the cytoplasm of neutrophils, later to be named ANCA (28). ANCAs have been then described for the first time in GPA patients in 1985 (29). Five years later, the proteinase 3 was identified as the ANCA antigen in GPA patients (30). The following section details clinical, *in vitro* and *in vivo* arguments supporting PR3-ANCA pathogenicity in GPA.

### Clinical Arguments of Proteinase 3-Anti-Neutrophil Cytoplasmic Antibody Pathogenicity

The first argument supporting the importance of PR3-ANCA in the pathogenicity of AAV is that classification of patients according to their ANCA specificity seems more relevant than the clinical presentation. Indeed, the presence of PR3-ANCA or MPO-ANCA correlates better with genetic factors, prognosis, and response to treatment than the clinical phenotype of GPA or MPA (31–36). This underlines the importance of the antigen recognized by ANCA (PR3 or MPO) in the disease and highlights the concept of PR3-AAV and MPO-AAV (31). A second argument, developed below, is the frequent and regular, but not strictly correlated fluctuations of PR3-ANCA levels with the activity of the disease (18–27). Furthermore, the efficacy of current therapies seeking to eliminate these auto-antibodies, such as plasma exchange, although recently challenged, and anti-CD20 therapy, supports the importance of PR3-ANCA in GPA (14, 26, 37, 38). Finally, a key indirect argument of PR3-ANCA pathogenicity is the fundamental role in the disease of neutrophils, their target cells: activated neutrophils are found in inflammatory tissue, vessel samples, and in the circulation of GPA patients (13, 39, 40).

### *In Vitro* Arguments of Proteinase 3-Anti-Neutrophil Cytoplasmic Antibody Pathogenicity

The most widely accepted pathophysiological hypothesis in GPA, although contested by some authors (41), assumes the central role of PR3-ANCA and neutrophils.

PR3-ANCAs are initially produced by B lymphocytes pointing to a loss of tolerance to PR3. Several hypotheses have been put forward to explain this acquired loss of tolerance. The first is that PR3 contained in neutrophil extracellular traps (NETs) during inflammatory responses is exposed to the immune system (39). This hypothesis is supported by the finding that neutrophils from GPA patients are more likely to produce NETs and tend to have a lower DNase I activity leading to a lack of clearance of NETs. Consequently, PR3 is exposed to antigen presenting cells on extracellular immune enhancing components of neutrophils, thereby breaking self-tolerance (39, 42, 43). The second hypothesis postulates that a deficiency in clearance of apoptotic neutrophils overexpressing membrane-bound PR3 (mbPR3) maintains a prolonged state of inflammation favoring the generation of anti-PR3 antibodies (44, 45). Indeed, the overexpression of mbPR3 inhibits efferocytosis, a mechanism involved in the elimination of apoptotic cells by M2 macrophages during the resolution phase of inflammation (44). The third hypothesis is that the production of autoantibodies is triggered during the course of an immune response against *Staphylococcus aureus* which is associated with GPA (46) or another pathogen (47). Indeed, it has been shown that some patients with PR3-ANCA also had antibodies directed against a peptide translated from the antisense DNA strand of PR3 (complementary PR3, cPR3) which included sequences from *Staphylococcus aureus*. In this study, immunization of mice with the middle region of cPR3 induced antibodies against cPR3 but also PR3, showing that auto-immunity can be initiated through an immune response against a peptide that is antisense or complementary to the autoantigen, which subsequently induces anti-idiotypic antibodies (48). Another argument for the triggering of auto-immunity by *Staphylococcus aureus* is its strong capacity to induce NET production by neutrophils (42). Hyper-reaction to influenza vaccine was also hypothesized in a case report to contribute to the development of AAV (49).

Once generated, PR3-ANCA binds to partially activated neutrophils primed *e.g.* by TNF alpha (TNFα) (50) and causes their excessive auto-immune activation responsible for vasculitis lesions (12, 13, 51). The widely accepted hypothesis of auto-immune activation of neutrophils by IgG ANCA was recently challenged by Popat and Robson (52). In their study, purified IgG from AAV patient sera, even in active disease, did not induce neutrophil activation. However, all experiments were performed on neutrophils obtained from only two healthy donors (52). Interestingly, in another study performed by the same group, the same IgG preparations obtained from MPO-ANCA positive patients promoted inflammation through monocyte stimulation but IgG preparations obtained from PR3-ANCA positive patients were not tested (53). Furthermore, excessive NET formation in AAV has been shown to be independent of the presence of ANCA while correlating with disease activity (54). Whatever, most of results found in literature are in favor of an auto-immune activation of neutrophils by PR3-ANCA.

**Figure 1:**
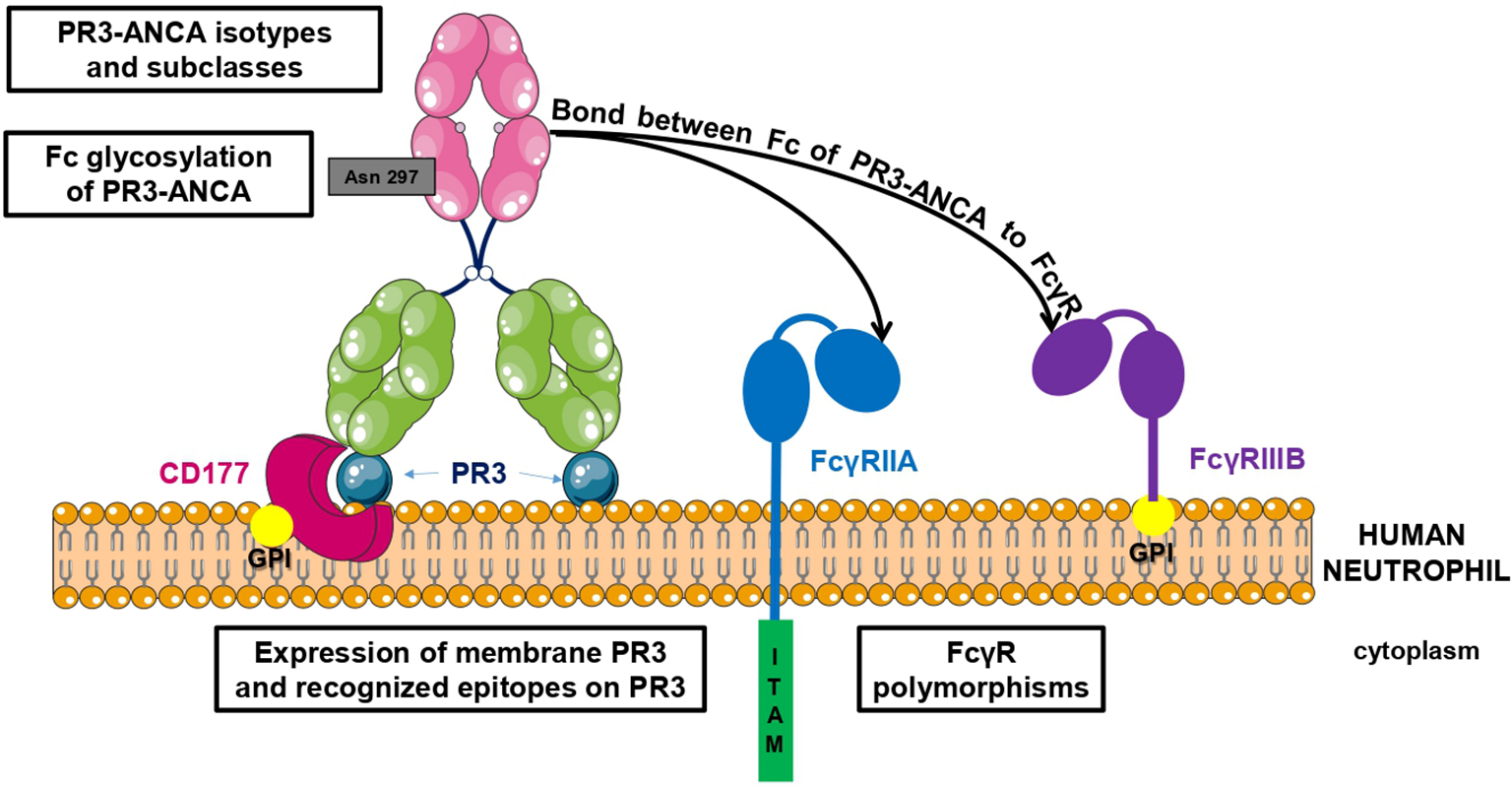
Factors involved in PR3-ANCA pathogenicity. There are two types of interaction between PR3-ANCA and neutrophils: one includes a link between the PR3-ANCA Fab and mbPR3 exposed at the surface of neutrophils and the other involves a bond between the Fc of PR3-ANCA and Fc*γ*R. Pathogenicity of PR3-ANCA depends on many factors including the expression of membrane PR3 on neutrophils, the recognized epitopes, the presence or not of Fc gamma receptor polymorphisms, the subclasses and isotypes of PR3-ANCA, and finally the Fc glycosylation of PR3-ANCA.

There are two types of interaction between PR3-ANCA, mainly involving IgG, and neutrophils: one includes a link between the PR3-ANCA Fab (fragment antigen binding) and mbPR3 exposed on the surface of neutrophils and the other involves a link between the Fc (fragment crystallizable) of PR3-ANCA and Fc gamma receptors (Fc*γ*R) (55, 56) (**Figure 1**). *In vitro* auto-immune activation of neutrophils by PR3-ANCA has been studied with human purified polyclonal PR3-ANCA or murine/chimeric anti-PR3 mAbs *in vitro* but never, to our knowledge, with human anti-PR3 mAb. Nevertheless, *in vitro* auto-immune activation of neutrophils by PR3-ANCA results in an adherent phenotype (57), induction of NETosis (39, 43), production of intra- and extra-cellular reactive oxygen species (ROS) (58–60), degranulation with protease release (PR3, elastase, cathepsin G) (58), actin polymerization in a calcium-dependent manner (55), and production of pro-inflammatory cytokines, in particular IL-8 (61, 62). The excessive release of all these mediators leads to a damage of the vascular endothelium and, therefore, to vasculitis lesions (63). This release of these mediators moreover triggers a vicious circle: NETosis contributes to tolerance failure, leads to PR3 exposure to extracellular DNA and activates the alternative pathway of the complement system (39, 42). In addition, IL-8 production and vascular damage enhance the recruitment of other inflammatory cells towards the inflammatory site (62).

### *In Vivo* Arguments of Proteinase 3-Anti-Neutrophil Cytoplasmic Antibody Pathogenicity From Murine Models

*In vivo* ANCA pathogenicity was demonstrated more consistently with MPO-ANCA than PR3-ANCA (64). The study of PR3-ANCA *in vivo* in a murine model is difficult and complex. Indeed, the hydrophobic patch allowing human PR3 exposure on the neutrophil surface is lacking in murine PR3, and therefore murine PR3 is not expressed on the neutrophil surface (65). Furthermore, murine and human PR3 are only 63% homologous (65). To overcome these differences, a research team generated a transgenic mouse model in which murine neutrophils expressed human PR3. Despite this, passive transfer of anti-PR3 antibodies failed to induce glomerulonephritis, partially because mice did not process human pro-PR3 into mature PR3 properly (66). Nevertheless, some studies have shown interesting results. In a model of acute lung injury, co-perfusion of human TNFα-primed neutrophils and monoclonal anti-PR3 antibodies induced pulmonary edema dependent on ROS production on isolated rat lungs (67). Moreover, in a humanized mouse model, administration of purified human IgG PR3-ANCA was able to partially reproduce pulmonary and glomerular lesions (68).

To conclude on GPA pathophysiology, despite the implication of other factors, such as the alternative pathway of the complement system, the regulatory cytokine network, monocytes, and T lymphocytes (69–72), PR3-ANCAs appear to play a fundamental role in GPA by inducing auto-immune activation of neutrophils. Therefore, understanding factors involved in the mechanism of auto-immune activation of neutrophils by PR3-ANCA is a necessary prerequisite to consider in the development of new biomarkers and therapies.

## Understanding Auto-Immune Activation of Neutrophils by Proteinase 3-Anti-neutrophil Cytoplasmic Antibody in Granulomatosis With Polyangiitis

Mechanisms of auto-immune activation of neutrophils by PR3-ANCA are influenced by several factors summarized in **Table 1** and illustrated in **Figure 1**. They have been divided into two groups depending on whether PR3-ANCA binds with the neutrophil on PR3 or Fc*γ*R.

**Table 1:** Factors influencing auto-immune activation of neutrophils by PR3-ANCA.

	Fab/PR3 interaction	Fc/Fc*γ*R interaction
PR3-ANCA characteristics	Epitope recognized on PR3 Cross-linking of PR3 Avidity for PR3 Affinity for PR3?	Isotype of immunoglobulin IgG subclasses Glycosylation on asparagine 297 Affinity for Fc*γ*RIIa and Fc*γ*RIIIb?
Neutrophil characteristics	% of mbPR3^+^ neutrophils Amount of mbPR3 Glycosylated isoforms of mbPR3	Polymorphism of Fc*γ*R Activation independent of Fc*γ*R engagement

Main consistent factors described in the literature are shown in bold.

### Factors Involved in mbPR3 and Proteinase 3-Anti-Neutrophil Cytoplasmic Antibody Interaction

#### Characteristics of mbPR3

PR3 is a 29-kDa serine protease which can be found in neutrophil granules and also exposed on the outer leaflet of the plasma membrane. This mbPR3 on the outer surface of neutrophils is the fraction recognized by Fab of PR3-ANCA. Two mbPR3 forms have been described: constitutive mbPR3 and induced-mbPR3 (5). Expression of the constitutive mbPR3 follows a bimodal distribution with mbPR3^+^ positive and negative neutrophils. The ratio between negative and positive neutrophil subpopulations varies extremely between individuals (ranging from 0 to 100%), but remains extremely stable throughout life (56, 73, 74). This bimodal distribution seems to be correlated with the distribution of neutrophil antigen B1 (NB1) also called CD177. NB1 is a glycosylphosphatidylinositol (GPI)-anchored neutrophil-specific membrane receptor which would serve as a co-receptor to allow PR3 expression on the neutrophil membrane (75, 76). Induced mbPR3 is due to a signal-dependent translocation of PR3 from granules to the membrane *e.g.* in response to TNFα stimulation. This TNFα priming is necessary for neutrophil activation by PR3-ANCA (60, 77). Nevertheless, constitutive mbPR3, devoid of enzymatic activity, is also recognized by PR3-ANCA (73). Furthermore, unlike induced-mbPR3, constitutive mbPR3 is not solubilized by alpha1-antitrypsin (A1AT), a natural protease inhibitor, and is therefore a permanent target for PR3-ANCA (78).

It was also suggested that PR3-ANCA could directly activate neutrophils by binding circulating soluble PR3 and forming a PR3-antibody immune complex (55), signifying that PR3-ANCA could bind to the neutrophil and activate them only by their Fc fragment. Another hypothesis concerning involvement of immune complex in AAV was reported by van Paassen et al. (79): ANCA antigens, after their release, will bind to the endothelial cell surface and tissue matrix and will then be bound by ANCA resulting in the formation of immune complexes. These immune complex deposits will enhance further recruitment and activation of neutrophils sustaining an innate inflammatory vicious circle (79). However, in a recent study, no immune complex deposits were found in the majority of renal biopsies of AAV patients whereas C3d, C4d, and C5b-9 were found in a majority of analyzed renal biopsies suggesting the implication of the alternative pathway of the complement system (80). The implication of the complement system in AAV, particularly the alternative pathway, has also been suggested by others (72).

#### Epitopes on PR3

Implication of epitopes recognized by PR3-ANCA on PR3 in GPA has been studied extensively. Different epitopes and epitope regions have been identified with polyclonal immunoglobulins from patients or with murine/chimeric anti-PR3 mAb but never with human anti-PR3 mAb. Most ANCAs clearly recognize conformational epitopes (81–83), but many more studies have been performed using linear peptides. Concerning epitope regions described with murine anti-human PR3 monoclonal antibodies, five epitopes have been described. Epitopes 1, 2, and 4 are located near the active site while epitope 3 is located very remotely on its posterior face and epitope 5 is found on the hydrophobic patch, allowing PR3 exposure at the neutrophil surface and thus rendering it inaccessible on the mbPR3 (84, 85). Results from studies describing the epitopes recognized during the active phase of the disease support this. With these studies, we can conclude that pathogenic PR3-ANCAs found in active disease have common characteristics: 1/they mainly bind PR3 close to its active site and close to the binding site of A1AT (84, 86, 87); 2/they have the capacity to modulate the enzymatic activity of PR3 *in vitro* (88–90) although the possible mechanisms of remote or direct interference remain to be clarified (91), and 3/they have the capacity to interfere with the complexation of PR3 with A1AT (87, 92). This interference with the complexation of PR3 with A1AT suggests that PR3-ANCA could have a direct pathogenic role through their Fab fragment (91). They could reduce the clearance of PR3 by A1AT allowing the prolongation of its inflammatory effects. They could also prolong its exposure to the immune system and perpetuate the vicious circle of auto-immunity. Decreased A1AT activity (93) and A1AT deficiency (94), observed in GPA, could act the same way in the disease process. The impact of PR3 glycosylation on its recognition by PR3-ANCA remains poorly studied. But it does not seem to be required (95, 96).

### Factors Involved in Fc Gamma Receptors and Proteinase 3-Anti-Neutrophil Cytoplasmic Antibody Interaction

#### Neutrophil Activation Mediated by FcyR

Neutrophil activation by PR3-ANCA mainly occurs after Fc domain binding with Fc*γ*R on the neutrophil surface. This neutrophil activation primarily leads to degranulation (58), ROS production (58–60), NETosis (39, 43), adhesion to endothelial cells (57), and secretion of pro-inflammatory cytokines, especially IL-8 (62). Neutrophils constitutively express Fc*γ*RIIa (CD32a) and Fc*γ*RIIIb (CD16b), which are both low-affinity and activating Fc*γ*Rs. These two receptors are not found in other species which also explains the difficulty to study GPA in animal models. Fc*γ*RIIa is a “classical” transmembrane Fc*γ*R with a cytoplasmic domain containing an immunoreceptor tyrosine-based activation motif (ITAM) domain. In comparison, Fc*γ*RIIIb, mainly expressed on neutrophils and on a minor subset of basophils, is an atypical GPI-linked receptor without the intra-cytoplasmic part, and thus it is not capable of intracellular signaling. The main hypothesis explaining the mode of action of Fc*γ*RIIIb is that this receptor colocalizes with other transmembrane receptors such as CD18 and Fc*γ*RIIa. Fc*γ*RI expression is induced after interferon gamma (IFN*γ*) stimulation and has been little studied in GPA (97–99).

Several characteristics of these Fc*γ*R and PR3-ANCA must be taken into account when trying to explain the modalities and consequences of the interaction between the Fc fragment of PR3-ANCA and Fc*γ*R of the neutrophil: the differential binding of immunoglobulin subtypes to the different Fc*γ*R leads to different neutrophil functions at varying intensities. Fc*γ*RIIa engagement induces increased L-selectin expression and is the predominant Fc*γ*R involved in phagocytosis, but this mechanism has not been identified as being involved in GPA (99–101). Fc*γ*RIIIb engagement induces actin polymerization in a calcium-dependent way, activation of *β*1-integrines, IL-8 secretion, and NET formation (98, 99). NETosis is an important phenomenon in the pathogenesis of auto-immune vasculitis and is induced particularly by ROS that are produced intracellularly after Fc*γ*RIIIb signaling (102–104). Nevertheless, it is still unclear which of these two Fc*γ*R is predominantly involved in neutrophil activation by PR3-ANCA and what role they play in ROS production. Some studies have shown a greater importance of Fc*γ*RIIa (105–107) while others state Fc*γ*RIIIb to be mainly involved (108). The hypothesis of a cooperation between these two receptors or with other surface neutrophil components such as complement receptor 3 (CR3) or *ß*_2_ integrins also seems relevant (97, 99, 109, 110). In the majority of studies, selective blocking of Fc*γ*RIIa or Fc*γ*RIIIb never abolished neutrophil activation completely suggesting that neither of these two receptors are exclusively responsible for neutrophil activation by PR3-ANCA (105, 111, 112). Therefore, the existence of a mechanism has been suggested by which PR3-ANCAs activate neutrophils in a manner distinct from conventional Fc*γ*R engagement. Direct stimulation of neutrophils with the Fab fragment of a PR3-ANCA was shown not to activate neutrophils whereas the Fab′_2_ fragment of PR3-ANCA, lacking the Fc domain, was able to induce moderate activation of neutrophils and triggered distinct signaling pathways (111, 113, 114). This indicates that cross-linking of the PR3 antigen, by Fab′_2_ PR3-ANCA could lead to neutrophil activation independent of signaling through Fc*γ*R.

Despite the importance of Fc*γ*R signaling in neutrophil activation, the association of Fc*γ*R polymorphisms with the disease remains unclear (115–117). In one study, the NA1 polymorphism of Fc*γ*RIIIb was associated with a higher NET production by neutrophils after *in vitro* PR3-ANCA stimulation and with the development of severe renal disease *in vivo* (118). The implication of NA1 polymorphism in MPO-AAV is an additional argument of its implication in AAV (115). On the other hand, a low FCGR3B copy number is associated with auto-immune diseases such as systemic lupus erythematosus (SLE), MPA, and GPA (119). Furthermore, the role of Fc*γ*R polymorphisms in GPA is supported by studies showing that patients homozygous for the Fc*γ*RIIA131H or Fc*γ*RIIIA158V alleles respond faster to immunosuppressive treatment with Rituximab and their disease progresses significantly faster than in other patients (120, 121).

#### Proteinase 3-Anti-Neutrophil Cytoplasmic Antibody Isotypes and Subclasses Involved in Neutrophil Activation

Among the human immunoglobulin (Ig) isotypes, IgA and IgM PR3-ANCAs have been found in GPA patients but remain little studied, and their implication remains controversial. IgA is found in a quarter of patients and in a small number of patients with severe renal impairment (118). IgM is found in 15 to 40% of cases, most often transiently (122). The isotype IgG, however, is the most frequent and most studied immunoglobulin isotype. IgG1 and IgG4 have been reported to be the most abundant IgG PR3-ANCA subclasses in sera from GPA patients (123–125). In general, IgG1 and IgG3, which can bind to Fc*γ*RIIa, are the major PR3-ANCA subclasses able to activate neutrophils. IgG3 seems to be the most pathogenic IgG PR3-ANCA subclass in GPA as they seem to play the greatest role in ROS production and IL-8 response, which subsequently leads to recruitment of other inflammatory cells and amplifies inflammation (62, 126). It should also be noted that IgG3 binds Fc*γ*RIIa and Fc*γ*RIIIb with a higher affinity than the other IgG subclasses (98). IgG2 PR3-ANCAs, which bind poorly or not at all Fc*γ*RIIIb (99), do not appear to have an important role in neutrophil activation in GPA. IgG4 has long been considered to weakly activate neutrophils because of its low affinity to Fc*γ*R. Furthermore, it is rather supposed to have an anti-inflammatory role partly due to a dynamic Fab arm exchange (127). Nevertheless, it was first shown that human polyclonal IgG4 PR3-ANCAs purified from patient sera were able to induce neutrophil activation (128). The same group then confirmed this result using a monoclonal chimeric IgG4 anti-PR3 antibody which was able to induce the release of superoxide, degranulation, and adhesion but not IL-8 secretion (101). Furthermore, they demonstrated, in this last study, that activation of neutrophil was dependent of Fc*γ*R engagement (101).

#### Particular Glycosylation of Proteinase 3-Anti-Neutrophil Cytoplasmic Antibody

The conserved glycosylation of asparagine 297 in the Fc domain of IgG is important for the interaction between IgG and Fc*γ*R. A modification of this glycosylation leads to a change of the Fc affinity towards Fc*γ*R and thus to an altered role in inflammatory processes (129–131). The presence and composition of this glycosylation seem to be fundamental in AAV. Indeed, enzymatic modification of IgG PR3-ANCA and MPO-ANCA glycans attenuates neutrophil activation (ROS production and degranulation) (132). Moreover, in the same study in a murine model of renal disease, complete deglycosylation of IgG MPO-ANCA induced by injection of the bacterial enzyme endoglycosidase S, led to a major decrease in the renal symptoms in these mice (132). This last result, only experimented for MPO-ANCA, should be tested for PR3-ANCA. Furthermore, modifications of this glycosylation are implicated in several auto-immune diseases, such as in GPA, as highlighted by Goulabchand et al. (133). IgG from GPA patients shows low levels of bisection, sialylation, and galactosylation in the active phase of the disease (134–138).

Therefore, the knowledge of these factors influencing PR3-ANCA pathogenicity, *i.e.*, their potential to induce auto-immune activation of neutrophils, could be exploited in view of improving GPA management (biomarkers and therapies) focused on the pathogenicity of PR3-ANCA.

## Applying Knowledge on Proteinase 3-Anti-neutrophil Cytoplasmic Antibody Pathogenicity

### Perspectives to Develop New Biomarkers

PR3-ANCA level combined with clinical manifestations provide insufficient results in predicting relapse (18–21, 23) except in patients with renal involvement, in whom this PR3-ANCA level correlates with disease activity (24, 25), and in patients after treatment with rituximab (26, 27). Furthermore, PR3-ANCA can persist in GPA patients during remission without predicting relapse (19). In a recent study of 126 patients, the utility of PR3-ANCA as a biomarker was examined. No strict clinical-immunological correlation was observed in 25% of the patients. PR3-ANCA remained positive in 21.7% of patients after the induction of treatment. Among patients with persistent PR3-ANCA, 27.4% did not relapse within 36–38 months, and 50% of them were in complete remission. Finally, 15% of patients in complete remission had persistently positive PR3-ANCA for more than 12 months (22). Therefore, GPA management cannot be based on PR3-ANCA level exclusively. It is essential to develop new biomarkers which could be used in combination as suggested by Osman et al. (139). Here, we have described several factors of PR3-ANCA that might influence their pathogenicity and which could be used as biomarkers. These factors are relevant either because of their difference between active disease and remission or because of their association with relapse. Although most of them are debated or insufficiently known, some of them are promising biomarkers: especially epitope specificities and the glycosylation pattern of PR3-ANCA.

The critical antigenic target of PR3-ANCA, *i.e.* mbPR3 expression, is important but has not been sufficiently investigated *in vivo* to be used as a biomarker of disease activity. Even though the percentage of mbPR3^+^ neutrophils was found to be higher in GPA patients than in healthy subjects and was correlated with the risk of relapse in some studies, other studies did not confirm this correlation (140, 141). The level of mbPR3 at the neutrophil surface correlates with ROS production and *in vitro* degranulation after stimulation of neutrophils with PR3-ANCA (142, 143). Nevertheless, only one study has highlighted that this level of mbPR3 changed during different stages of the disease and correlated with disease activity (144).

Contrary to MPO-ANCA associated vasculitis, in which a linear epitope (aa447–459) is exclusively associated with active disease (83), there is no epitope specifically pointing to disease activity in GPA. Nevertheless, these results obtained with MPO-ANCA allow the hope that epitopes associated with disease activity could be found in PR3-AAV. Several results, however, underline the importance of epitope-specificities of PR3-ANCA to determine their pathogenicity. Concerning epitopes targeted by PR3-ANCA in the active phase of the disease, they have common consistent characteristics which could be useful biomarkers. Indeed, pathogenic PR3-ANCAs: 1/bind PR3 close to the active site of PR3 (84, 86, 87), 2/inhibit the enzymatic activity of PR3 *in vitro* (88–90), and 3/have the capacity to interfere with the complexation of PR3 with its natural inhibitor A1AT (87, 92). Therefore, these common characteristics could be helpful in the diagnosis of the active form of the disease. Furthermore, they could enable a GPA patient and a healthy person with a positive PR3-ANCA result to be differentiated: PR3-ANCAs found in healthy donors target different epitopes than those found in GPA patients (86). Furthermore, it has been demonstrated that PR3-ANCAs target epitopes on PR3 with different proportions between patients and with a different evolution in the same patient according to the state of disease activity (145, 146). Therefore, studying epitope shift during patient follow-up seems promising in predicting relapse. To date, epitope shift has been associated with relapse in one prospective study: among 12 patients with relapse, an epitope shift was observed in 11 cases from epitopes located in the C-terminal towards epitopes in the N-terminal part of PR3. Furthermore, in the same study, the relapse rate was significantly higher in the group of patients with predominantly C-terminal reactivity at diagnosis compared to the group with N-terminal reactivity (147).

Only one study has investigated the influence of interaction strength between PR3 and PR3-ANCA and found a correlation between the avidity of this interaction and relapses in patients with renal impairment (148). The affinity of PR3-ANCA to PR3 could also influence their pathogenicity but has never been investigated to our knowledge.

As described above, most isotypes of Ig, at different levels, appear to be involved in GPA and are able to activate neutrophils. IgG is generally studied *in vitro*;IgG1 and mostly IgG3 are the two main IgG subclasses able to induce neutrophil activation (62). In one study, respiratory burst induced by IgG fractions from patients correlated with the disease activity and was related to changes in the relative amount of the IgG3 subclass of PR3-ANCA (126). Nevertheless, measurement of IgG3 subclass of PR3-ANCA did not improve the predictive value of a rise in ANCA in another study (18). Clinical implications of different Ig isotypes in disease activity, however, are studied separately and provide few or contradictory results, offering no consistent data for use as biomarkers. IgG4:IgG RNA ratio, representing the number of IgG4-producing B-lineage cells, seems to significantly differentiate active disease from remission (149). One study postulated that IgA PR3-ANCA had a protective role (118), whereas in another, their level was related to disease activity (150). IgM has been associated with the severity of the disease, particularly in severe pulmonary impairment (122). In contrast, other authors proposed a protective role of IgM as it can be found in healthy donors and patients in remission (151). Furthermore, a reduced frequency of marginal zone-like B cells, which are the main producers of IgM, has been observed in the circulation of patients with auto-immune vasculitis (152). Therefore, the role of isotypes and subclasses of Ig in GPA pathophysiology requires further investigation. They could be relevant biomarkers as they can be studied easily in the laboratory.

Glycosylation of total IgG and IgG PR3-ANCA could also be used as relevant biomarker. Indeed, IgG1 from GPA patients in the active phase of the disease shows low levels of bisection, sialylation, and galactosylation (134–138) and clinical remission was associated with glycan normalization in one study (138). Espy et al. highlighted that hyposialylation of IgG PR3-ANCA was correlated with disease activity and with the *in vivo* production of ROS by neutrophils (135). Therefore, studying ANCA glycosylation, particularly sialylation, of total IgG and IgG PR3-ANCA could be helpful to evaluate disease activity and probably to affirm remission.

An interesting biomarker could be the PR3-ANCA producer cell itself. Cornec et al. found a higher proportion of PR3-specific B cells among patients with active disease (2.91%) than among patients in remission (0.99%), whereas there was no difference in serum PR3-ANCA levels between the two groups (153). Studying the global B-cell population after a remission induction treatment with rituximab also seems attractive in GPA patients. Indeed, B-cell repopulation was associated with relapses whereas their absence predicted a relapse-free status (27).

To further investigate PR3-ANCA pathogenicity, we developed in our laboratory human anti-PR3 mAbs obtained after immortalization of memory B cells from GPA patients at different stages of the disease. We obtained an original anti-PR3 mAb (called 4C3) from a GPA patient in remission having a persistently high PR3-ANCA level. Neutrophil stimulation with 4C3 did not induce auto-immune activation of neutrophils *in vitro*, which demonstrates for the first time that non-pathogenic PR3-ANCAs exist. The existence of non-pathogenic PR3-ANCA, as 4C3, must be confirmed by further studies. However, their existence could explain why PR3-ANCA can persist in some GPA patients in remission without predicting relapse (22) and also why they can be found in healthy people (10, 11). Furthermore, due to the absence of any functional defect in its Fc fragment, we hypothesize that its non-pathogenic character is related to the epitope recognized on PR3. Indeed, mAb 4C3 binds mbPR3 on a newly described epitope close to the hydrophobic patch (154). Epitopes associated with non-pathogenicity of PR3-ANCA could be a relevant biomarker.

### Perspectives to Develop New Therapies

Despite current therapies, GPA is still a serious disease with an important mortality rate (15, 155), a significant morbidity related to the disease and its management (16) and a high risk of relapse (17). Furthermore, none of the treatments used or studied in research (51) are specific to the fundamental auto-immune activation of neutrophils by PR3-ANCA. The most recently used treatment is the application of rituximab, a chimeric monoclonal anti-CD20 antibody that depletes circulating B lymphocytes without specifically targeting those producing ANCA. This depletion is not immediate, with a decrease in ANCA of just 50% after one month (156), and includes several side effects such as significant risk of hypogammaglobulinemia, infection, lymphopenia, and neutropenia (156–159). Therefore, it seems necessary to look for more specific treatments for GPA which specifically block auto-immune activation of neutrophils by PR3-ANCA. Blocking this interaction could directly neutralize the effector cells more targeted and potentially suppress the inflammatory cascade faster. This new therapeutic approach could reduce the morbidity related to side effects of non-specific treatments currently used and also the morbidity related to complications of inflammation due to the disease.

Different treatment options could be considered to achieve this purpose. Elimination of PR3 autoantigen has already been proposed by Korkmaz et al. with promising results for a cathepsin C inhibitor (160, 161). Cathepsin C is a central biosynthetic switch in the activation of many serine proteases in immune cells and is responsible for the maturation of pro-PR3 to active PR3 (161). Consequently, using a cathepsin C inhibitor could lead to a significant decrease in the level of both PR3 autoantigen and PR3-ANCA (162, 163). Moreover, inhibition of neutrophil activation by PR3-ANCA has already been demonstrated *in vitro* by neutrophils pre-incubated with A1AT (78, 164, 165). Nevertheless, A1AT has the disadvantage of not being very sensitive for PR3 (85) and not eliminating constitutively expressed mbPR3 making its clinical use difficult (78). In our laboratory, we chose to develop full human mAbs targeting PR3 by immortalizing B cells from patients with GPA. Compared to a small chemical molecule, an antibody has several advantages: a larger target binding area for better specificity, a greater ability to hide the target, and a longer half-life (166).

Considering all these aforementioned factors influencing the auto-immune activation of neutrophils by PR3-ANCA, it would be interesting to find an anti-PR3 mAb to neutralize this interaction on condition that it has the following characteristics: it should bind to mbPR3 with a high affinity and target the major epitopes of PR3, without binding to the “hydrophobic patch” which is not accessible on mbPR3, in order to prevent the fixation of the majority of pathogenic PR3-ANCA (84, 85). The antigenic moiety, essentially of mbPR3, should also be taken into account in the dose used because of its variation during the course of the disease (55). It should obviously not induce neutrophil activation.

Several strategies could be used to create an anti-PR3 mAb that does not activate neutrophils. The majority of these strategies aim to block Fc*γ*R engagement. A first avenue would be to keep the mAb in its complete form which would give it the advantage of having a longer half-life. Modification of its glycosylation could be a solution as IgG glycosylation is important in Fc*γ*R engagement (129, 138). Interestingly, in a murine model it has been shown that when MPO-ANCA is deglycosylated, *in vitro* neutrophil activation and *in vivo* vasculitis symptoms decrease significantly (132). Whatever, PR3-ANCA deglycosylation has never been studied *in vivo*. Changing the subclass, for example using an anti-PR3 IgG2 or IgG4 recombinant antibody seems to be risky. These two isotypes have weak affinities for Fc*γ*R expressed by neutrophils (98, 99), but still seem to be involved in the pathophysiology of GPA as anti-PR3 IgG4 has already been shown to be able to activate neutrophils *in vitro* (101, 128). Another strategy would be to use antibody fragments. Fab fragments of PR3-ANCA seem to be interesting because they were able to bind mbPR3 without causing neutrophil activation in most studies, while Fab′_2_ fragments of PR3-ANCA did, probably through cross-linking the PR3 antigen (111, 113, 114). Despite this, it has the disadvantage of being rapidly eliminated because of its low molecular weight. To overcome this, a multi-specific recombinant Fab with an extended half-life could be a solution. Moreover, a combination of several different Fab fragments targeting different epitopes on the mbPR3 could be used in order to prevent the majority of circulating pathogenic PR3-ANCA from binding with neutrophils.

Finally, we hypothesize that a human anti-PR3 mAb could directly prevent neutrophils from activating. As described above, we have produced the mAb 4C3, a non-pathogenic human anti-PR3 mAb which was shown to be unable to activate neutrophils *in vitro.* Moreover, this mAb is able to neutralize auto-immune activation of neutrophils induced by pathogenic PR3-ANCA from GPA patients at diagnosis (154). This promising result offers perspectives to develop new therapies in GPA but must be confirmed by further studies.

## Concluding Remarks

GPA is a rare but serious auto-immune vasculitis in which auto-immune activation of neutrophils, enabled by their interaction with PR3-ANCA, plays a central role. GPA management (monitoring and treatment) could be improved based on a better knowledge of factors influencing PR3-ANCA pathogenicity. Therefore, a better understanding of these factors and the confirmation of the existence of non-pathogenic PR3-ANCA could lead to developing new potential biomarkers, such as paratope and glycosylation of PR3-ANCA. Indeed, pathogenic characteristics and total level of PR3-ANCA could be useful biomarkers to evaluate disease activity, to predict relapse in GPA patients, and to differentiate a GPA patient and a healthy person with positive PR3-ANCA. Furthermore, targeting PR3-ANCA interaction with neutrophils, especially with monoclonal antibodies or antibody fragments, seems to offer an attractive perspective and could represent a more focused therapeutic, thereby avoiding overtreatments and achieving higher efficacy with fewer side effects than current therapies.

## Author Contributions

JG, RL, DN, SW, DJ, BK, and CH prepared and wrote the manuscript. All authors contributed to the article and approved the submitted version.

## Funding

This work was supported by the recurrent annual financial support from the University of Tours and by a grant from Labex MabImprove. This work was supported by Région Centre-Val de Loire through the program Ambition Research and Development «Biopharmaceuticals». JG is a recipient of a master degree grant (Année Recherche) from the Hospital of Tours. RL, DN, and CH are supported by the University of Tours. BK is supported by INSERM. DJ and SW are supported by the Max-Planck-Institute of Neurobiology and by the European Union’s Horizon 2020 research and innovation program under Grant 668036 (RELENT).

## Conflict of Interest

The authors declare that the research was conducted in the absence of any commercial or financial relationships that could be construed as a potential conflict of interest.
